# Clinical and genetic diagnostic challenges in presumed hereditary ataxia

**DOI:** 10.1007/s00415-026-13756-7

**Published:** 2026-03-23

**Authors:** Helene Faust, Patricia Duffek, Stephan Drukewitz, Janina Gburek-Augustat, Petra Baum, Christa-Caroline Bergner, Steffen Syrbe, Julian Schröter, Rami Abou Jamra, Denny Popp

**Affiliations:** 1 Institute of Human Genetics, University Hospital Schleswig-Holstein, Arnold-Heller-Str. 3, 24105 Kiel, Germany; 2 Institute of Human Genetics, University Medical Center Leipzig, Philipp-Rosenthal-Str. 55, 04103 Leipzig, Germany; 3 Division of Neuropediatrics, Hospital for Children and Adolescents, University Hospital Leipzig, Leipzig, Germany; 4 Department of Neurology, University Hospital Leipzig, Leipzig, Germany; 5 Division of Pediatric Epileptology, Clinic 1, Medical Faculty of Heidelberg, Center for Child and Adolescent Medicine, Heidelberg University, Heidelberg, Germany

Dear Sirs,

Cerebellar ataxia is present in many different genetic disorders, such as autosomal-dominant spinocerebellar ataxias (SCA), autosomal-recessive ataxias and mitochondriopathy [1]. With the growing use and possibilities of genetic diagnostics, many overlaps between neurogenetic disorders and highly variable clinical manifestations have been identified. For instance, many genes that were previously assigned to either hereditary ataxia or hereditary spastic paraplegia (HSP) have now been associated with both ataxia and HSP [2]. In line with clinical heterogeneity, the genetic causes of hereditary cerebellar ataxia are manifold. Genetic alterations include single nucleotide variants (SNVs), copy number variations (CNVs), structural variants (SVs), mitochondrial DNA (MT-DNA) variants and frequently short tandem repeat (STR) expansions [3]. SNVs, CNVs and, if included, MT-DNA variants can be readily detected in parallel using diagnostic state-of-the-art sequencing: short-read whole exome (WES) and whole genome sequencing (WGS) [4, 5]. In contrast, STR expansions are more challenging to detect due to the limitation of short-reads to 100-150 bp as many repeat expansions exceed this length by far. Nonetheless, bioinformatic tools have been developed to enable the detection of repeat expansions in short-read WGS data [6]. Several studies demonstrated the benefit of repeat expansion detection with ExpansionHunter also in WES data for loci in or near coding regions [7, 8]. However, due to limited read length, short-read sequencing can usually only be used as a screening test for repeat expansion detection and a second method is necessary. In contrast, long-read sequencing (LRS) allows accurate and comprehensive genotyping of repeat expansions and identification of methylation changes [9].

We applied these new diagnostic possibilities to identify the diagnosis in eight patients with cerebellar ataxia, a positive family history, and a negative prior WES analysis (see Online Resource 1 for details especially Table S1). Therefore, we performed re-evaluation of WES data, including MT-DNA and repeat expansion analysis, short-read WGS including repeat expansion analysis and long-read WGS to confirm repeat expansions and expand the genetic diagnostics (see Fig. 1).

**Fig. 1:**
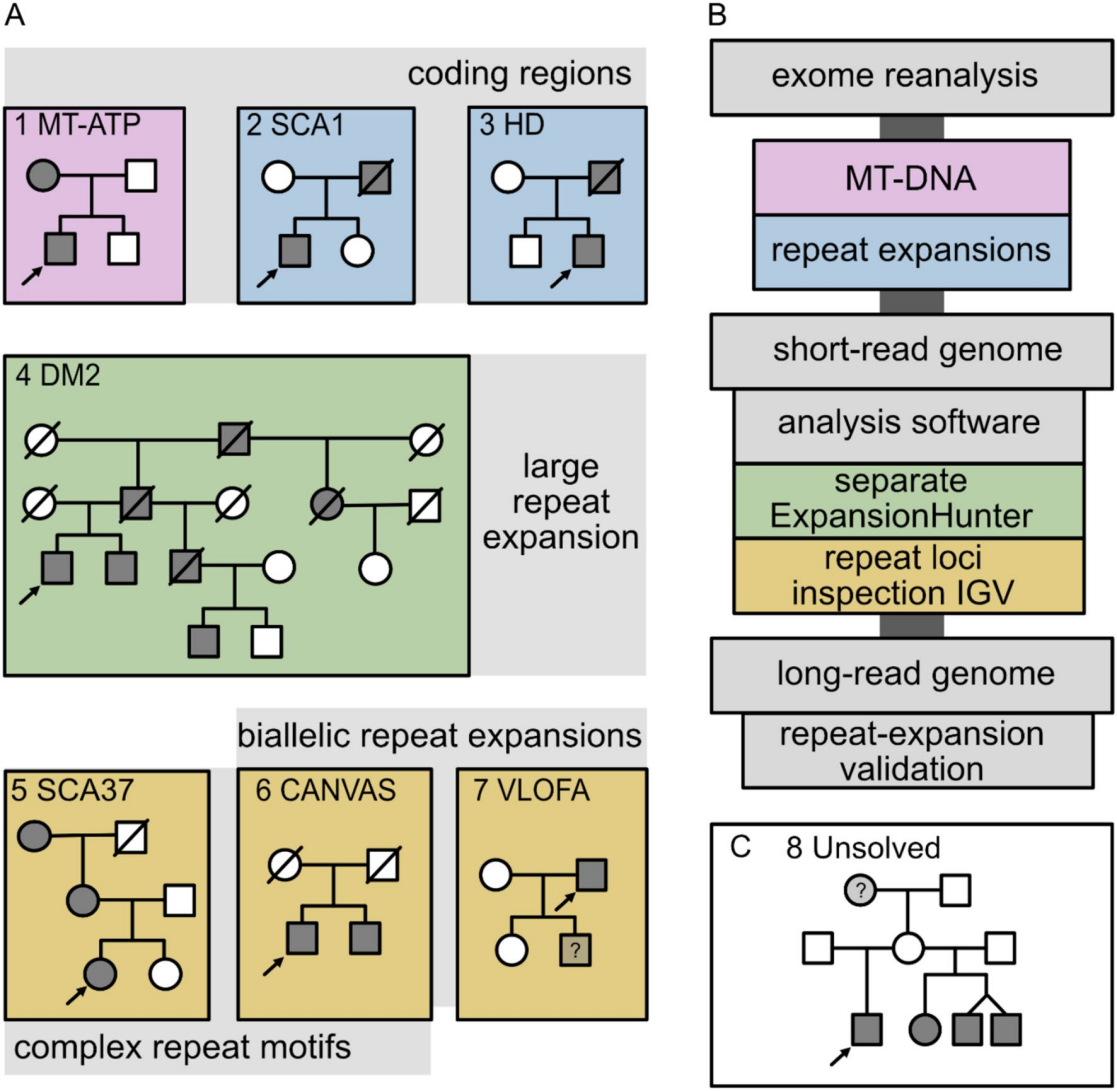
**A** Pedigrees of patient 1–7 and identified diagnoses, colored according to the analysis resulting in the diagnosis, **B** analysis workflow, **C** pedigree of patient 8 without diagnosis

Patient 1 was a 14-year-old male with slowly progressive peripheral neuropathy and cerebellar ataxia with an age of onset at seven years. The peripheral neuropathy was manifested in leg-dominantly distal muscle weakness and pes cavus. Electrophysiology revealed an axonal and demyelinating motor neuropathy. Additionally, he had a learning disability and a horizontal gaze-evoked nystagmus and cerebellar atrophy in cerebral MRI. His mother had a sensorimotor leg-dominantly polyneuropathy and a progressive gait disturbance with spastic ataxic gait and paraparesis with age of onset at 16 years. Re-evaluation of trio exome and the additional annotation of MT-DNA variants revealed the pathogenic variant m.9185T>C, p.(Leu220Pro) in the gene *MT-ATP6* homoplasmic in patient 1 and heteroplasmic (98 %) in the mother. The variant was classified as pathogenic [10] and hence, the diagnosis of a *MT-ATP6*-associated mitochondriopathy was confirmed in patient 1 and his mother.

Patient 2 was a male who developed ataxia, frontocerebellar atrophy and axonal sensory polyneuropathy at the age of 45 years. His father showed gait disturbance since the age of 30 years and was wheelchair-bound since the age of 49 years. Repeat analysis of WES data showed a monoallelic expansion to 47 CAG repeats in the gene *ATXN1* without interruptions. It has to be noted that previously a homozygous unremarkable repeat count was detected by targeted testing of the gene *ATXN1 based on PCR fragment analysis using capillary electrophoresis*. Nanopore genome sequencing revealed a heterozygous pathogenic expansion of 44 CAG-repeats in the gene *ATXN1* free of CAT interruptions and confirmed the diagnosis of SCA1.

Patient 3 was a male who developed tremor and ataxia at the age of 29 years. His father had an unknown movement disorder and was deceased. In this study, repeat analysis of WES data revealed a heterozygous pathogenic repeat expansion of 50 CAG-repeats in the gene *HTT*. Nanopore sequencing showed a monoallelic repeat expansion to 47-52 CAG-repeats, indicating some somatic variability. A retrospective phenotyping one year after initially genetic diagnostics revealed dysarthria, ataxia, abnormal saccadic eye movements, ptosis, impaired ocular adduction, rigor, bradykinesia, hypokinesia and cognitive impairment, and was consistent with the diagnosis of Huntington’s disease (HD).

Patient 4 was a male with suspicion of SCA due to ataxia and cognitive decline. Age of onset was at 55 years. His brother, father, paternal grandfather, paternal half-sister of the father, paternal half-brother and son of paternal half-brother were similarly affected. Separate repeat expansion analysis with ExpansionHunter of WGS data resulted in hints for a heterozygous repeat expansion to at least 839 CCTG repeats in the gene *CNBP*. In contrast, included repeat expansion analysis in the analysis software showed only 37 CCTG repeats in *CNBP* on the larger allele. Nanopore genome sequencing revealed a monoallelic pathogenic repeat expansion of 1355-1364 CCTG repeats with some interruptions by other motifs in the gene *CNBP*. Neurological examination showed a mild leg-dominantly proximal muscle weakness, flaccid dysarthria, cognitive impairment, hypomimia and no cerebellar ataxia. Electromyography showed myopathic features. Non-neurological symptoms were cataract, hearing impairment and cardiac events. In view of the new neurological examination results and the other symptoms. the diagnosis of myotonic dystrophy type 2 (DM2) was also confirmed clinically.

Patient 5 was a 48 year-old female with a very slowly progressive cerebellar ataxia characterized by gait ataxia, vertical and horizontal gaze-evoked nystagmus, dysarthria and cerebellar atrophy. She recognized the first symptoms at the age of 35 years. The mother and the maternal grandmother showed similar symptoms. In WGS a repeat expansion of 38 ATTTT/ATTTC repeats in the gene *DAB1* was determined in patient 5 and her mother by ExpansionHunter included in commercial software and separate analysis, but the exact motif remained elusive. An evaluation of the repeat region using IGV showed the pathogenic motif ATTTC. Nanopore genome sequencing confirmed a repeat expansion as insertion of 97 pathogenic ATTTC-repeats flanked by 141 ATTTT-repeats downstream and of 10 ATTTT-repeats upstream of the ATTTC motif in the gene *DAB1* and hence, the diagnosis of a SCA37.

Patient 6 was a 70 year-old male who showed ataxia, vertigo and cerebellar atrophy since the age of 55 years. His brother had similar symptoms. Short-read WGS using the analysis software and the separate ExpansionHunter analysis were unremarkable. However, manual inspection of the *RFC1* repeat locus in IGV indicated the pathogenic AAGGG repeat motif in all reads and, therefore, suspicion of biallelic repeat expansion in *RFC1*. Nanopore genome sequencing confirmed a biallelic repeat expansion as insertion of 862-891 and 1010-1159 pathogenic AAGGG repeats in the *RFC1* gene. Neurological examination showed disturbance of gaze stabilization (horizontal gaze nystagmus, down-beat nystagmus, saccadic eye movements), cerebellar ataxia, impaired vestibulo-ocular reflex and vestibulo-ocular suppression. Cranial MRI revealed cerebellar atrophy and electrophysiology, a sensory neuropathy, consistent with the diagnosis of cerebellar ataxia, neuropathy, vestibular areflexia syndrome (CANVAS).

Patient 7 was a 54-year old male with spastic-ataxic gait and vertigo with age of onset at 44 years. The patient indicated his son showed a mild ataxia which, however, was not examined within this study. In short-read WGS, both repeat expansion analysis included in the analysis software and separate ExpansionHunter calculation indicated a monoallelic repeat expansion of 92 and 364 GAA-repeats, respectively, in the gene *FXN*. Manual inspection in IGV revealed a repeat count larger than the read length in all reads and the suspicion of a biallelic repeat expansion in *FXN*. Long-read sequencing confirmed a biallelic repeat expansion of 97 to 122 (median 111) and 773 to 1088 (median 883) GAA-repeats and the diagnosis of very-late-onset Friedreich’s ataxia (VLOFA). Neurological investigation showed mild ataxia, spasticity, mild dysarthria, mild sensorimotor polyneuropathy and central afferent and efferent disorder. Additionally, the patient reported a hand tremor that could be clinically interpreted as an essential tremor.

Patient 8 was a 12-year-old male with spastic-ataxic gait disturbance, vertigo and intermittent hand tremor with age of onset at 4 years and mild developmental delay. His eight years old maternal half-sister and seven years old maternal half-brothers, who are identical twins, showed similar symptoms. The brain MRI revealed a cerebellar atrophy and supratentorial deficits in the half-sister. The maternal grandmother showed features of parkinsonism. The mother has no signs of a movement disorder. Exome analysis, including MT-DNA analysis of the patient, his half-sister and one of his half-brothers remained unremarkable. Re-evaluation of exome data, including repeat expansion analysis, short-read as well as long-read genome sequencing, remained unremarkable and the cause of the symptoms is still unclear. Therefore, sequencing data will be re-evaluated in a timely manner.

The diagnosis in seven of eight patients with presumed hereditary ataxia and previously unremarkable exome analysis was identified by re-evaluation of exome data or short-read genome sequencing, including comprehensive repeat expansion evaluation. Additionally, repeat expansions were confirmed by long-read sequencing.

Diagnoses were previously missed due to technical limitations or difficult clinical interpretation. In case of identified *MT-ATP6*-associated mitochondriopathy, MT-DNA analysis was not included in the first exome evaluation and a mitochondriopathy was clinically not suspected. Indeed, mitochondriopathies present clinical and diagnostic challenges due to highly variable clinical manifestations [11]. The benefits of including MT-DNA analysis in exome sequencing for the diagnostics of neurogenetic disorders have already been demonstrated in several studies [4, 5, 12]. Hence, MT-DNA analysis is advisable for exome diagnostics—and likewise genome diagnostics—even though a mitochondriopathy is not suspected in the first place.

Furthermore, diagnoses of HD, DM2 and VLOFA as presented here were also clinically not suspected, but confirmed by retrospective phenotyping as well. Patient 3 with identified HD presented with ataxia and tremor at initial diagnostics, but with characteristics of HD at retrospective phenotyping one year later. Franklin et al. [13] observed cerebellar ataxia as a relevant symptom in HD patients, especially in early disease stages. Cerebellar Purkinje cell loss in HD patients with predominantly motor symptoms was shown in several studies supporting cerebellar involvement in HD.

[14]. Hence, based on the case presented here as well as on descriptions in the literature, analysis of a repeat expansion in *HTT* should be considered for patients with cerebellar symptoms as well.

Patient 4 diagnosed with DM2 was referred for genetic testing due to ataxia and cognitive decline. However, retrospective phenotyping (many years after the first genetic diagnostics) did not reveal ataxia, but mild muscle weakness and myopathic features in electromyography. We cannot conclude whether a mild muscle weakness mimicked ataxia or what else led to the impression of ataxia in the first neurological examination. However, the clinical diagnoses of DM2 could be challenging as neurological symptoms can be mild, and multisystemic manifestations, as cataract and cardiac conduction defects, are variable [15]. Hence, all symptoms besides neurological manifestations should be taken into account for genetic diagnostics and phenotyping in the course is recommended, especially when initial testing was inconclusive.

There was also no suspicion of the disease in patient 7 with VLOFA, who was referred to a genetic diagnostic due to spastic-ataxic gait and vertigo. This was one of two patients included in this study who did not receive targeted repeat expansion testing. However, the clinical diagnosis of delayed-onset Friedreich ataxia (FA), including late-onset FA and VLOFA, is challenging due to variable symptoms and differences compared to typical FA. Lecocq et al. [16] observed that patients with delayed-onset FA show, compared to typical FA a lack of severe dysarthria, more frequent retained tendon reflexes, less frequent amyotrophy, muscle weakness, extensor plantar reflex, axonal sensory neuropathy, and cerebellar atrophy, as well as less frequent extra neurological symptoms. Spastic ataxia was observed as a first symptom in delayed-onset FA [16].

The cases with mitochondriopathy, HD, DM2 and VLOFA, respectively, demonstrate the challenging interpretation in presumed hereditary ataxia. In addition to the spastic-ataxic spectrum and mitochondriopathies [2, 11], a wide phenotypic variability is increasingly found in repeat expansion disorders. On the one hand, there were observed other neurodegenerative phenotypes in ataxia-associated repeat expansion disorders, such as Parkinsonism in SCA2, SCA3, SCA6, SCA8 and SCA17 or pure sensory neuropathy in *RFC1*-related disorders [17, 18]. On the other hand, ataxia is reported to be a feature in non-ataxia-associated repeat expansion disorders, as in HD [13]. Hence, we should think beyond disease groups in genetic diagnostics of neurodegenerative diseases.

In the case of patient 5 with SCA37 and patient 6 with CANVAS, the described phenotype fit perfectly to the patients’ symptoms, but both diseases were not yet described at the time of the first evaluations. Both cases demonstrate the benefit of reassessing differential diagnoses in the disease course and of re-evaluating genetic diagnostics. As the number of newly discovered repeat expansion disorders is continuously growing and encompass also frequent disorders, such as SCA27B [19], re-analyses and, if needed, expansion of genetic diagnostic is becoming increasingly crucial.

In contrast, in patient 2 with SCA1 the disease was suspected and targeted testing was performed, but was not identified due to a homozygous signal for the repeat expansion in *ATXN1*. This is an example for the advantage of NGS compared to targeted PCR-based repeat analysis with the possibility of an allelic dropout resulting in a homozygous signal.

In one family, we did not identify the cause of the symptoms, despite extensive analysis. 29 to 71% of individuals with ataxia remain without a genetic diagnosis [20], depending on the structure of the cohort. The diagnostic yield is considerably higher for patients with early onset and a positive family history, as analyzed here, than for sporadic cases with later onset [21]. Reasons for the diagnostic gap likely include unknown genotype–phenotype associations, technical limitations, and unidentified polygenic factors.

In conclusion, these eight cases demonstrate clinical and genetic diagnostic challenges in suspected hereditary ataxia, or rather in neurodegenerative diseases in general. The high variability of clinical presentations in neurodegenerative disorders implies thinking beyond specific disease groups in genetic diagnostics and performing comprehensive genetic testing that enables the detection of newly discovered genetic disorders. However, there are still limitations of comprehensive genome sequencing. Short-read WGS with bioinformatic tools cannot reliably detect all genetic alterations, especially not all kinds of repeat expansions (see Supplementary Material for an extended discussion), and long-read WGS is yet expensive and not available in most diagnostic laboratories. Therefore, thorough clinical assessment, longitudinal phenotyping, and periodic re-evaluation of genetic data remain essential to integrate new genotype–phenotype discoveries.

## Supplementary Information

Below is the link to the electronic supplementary material.

Online Resource 1: Supplementary methods and extended discussion on repeat expansion detection in short-read sequencing data. Supplementary file1 (PDF 353 KB)

Online Resource 2: Variant catalog based on gnomAD information with offtargets (genome built GRCh37). Supplementary file2 (JSON 82 KB)

Online Resource 3: Variant catalog based on gnomAD information with offtargets (genome built GRCh38). Supplementary file3 (JSON 86 KB)

Online Resource 4: Thresholds for pathogenic STR expansion. Supplementary file4 (XLSX 14 KB)

Online Resource 5: IGV screenshots of relevant STR expansions. Supplementary file5 (PDF 900 KB)

## Data Availability

Data in pseudonymized form supporting the findings of this study are available upon reasonable request to the corresponding author.
