# Supplementary material for: Clinical and genetic diagnostic challenges in presumed hereditary ataxia
Source: J Neurol. 2026 Mar 23;273(4):225. doi: 10.1007/s00415-026-13756-7 (PMC13009057; doi:10.1007/s00415-026-13756-7)
Supplement: Supplementary file 1 — Online Resource 1: Supplementary methods and extended discussion on repeat expansion detection in short-read sequencing data. Supplementary file1 (PDF 353 KB) [file 415_2026_13756_MOESM1_ESM.pdf]

Supplementary Information for

CLINICAL AND GENETIC DIAGNOSTIC CHALLENGES IN PRESUMED HEREDITARY ATAXIA

Helene Faust, MD[1,2*], Patricia Duffek[2], Stephan Drukewitz, PhD[2], Janina Gburek-Augustat, MD[3], Petra Baum, MD[4], Christa-Caroline Bergner, MD[4], Steffen Syrbe, MD[5], Julian Schröter, MD[5], Rami Abou Jamra, MD[2], and Denny Popp, PhD[2*]

[1]Institute of Human Genetics, University Hospital Schleswig-Holstein, Kiel, Germany
[2]Institute of Human Genetics, University Medical Center Leipzig, Leipzig, Germany
[3]Division of Neuropediatrics, Hospital for Children and Adolescents, University Hospital Leipzig, Leipzig, Germany
[4]Department of Neurology, University Hospital Leipzig, Germany
[5]Heidelberg University, Medical Faculty of Heidelberg, Center for Child and Adolescent Medicine, Clinic 1, Division of Pediatric Epileptology, Heidelberg, Germany

Supplementary methods

Study concept

We identified 25 patients with the suspicion of a hereditary ataxia and a positive family history, that underwent exome sequencing at the Institute of Human Genetics in Leipzig between 2017 and 2023, and remained without a diagnosis. Of these 25 patients, eight patients could be enrolled for this study. All participants previously received WES as singletons or combined with affected family members that remained unremarkable. Six of the patients also underwent comprehensive targeted testing without identifying a diagnosis (see Table S1). First, WES data were re-evaluated and complemented by MT-DNA analysis, if these were not available at the time of the initial WES analysis. Additionally, an STR expansion analysis was performed using ExpansionHunter. Second, short-read WGS was carried out for all remaining cases with inconclusive exome sequencing. WGS evaluation was performed with commercial analysis software including ExpansionHunter analysis and additionally repeat expansion analysis with separate ExpansionHunter analysis. In addition, repeat expansion loci were visually inspected using IGV version 2.16.2 RRID:SCR_011793 (Integrative Genomics Viewer) [1]. Nanopore genome sequencing was performed if there were indications of repeat expansions or in case of unremarkable short-read WGS (see Figure 1B).

DNA extraction

Genomic DNA from peripheral blood leukocytes was isolated using the MagCore® HF16 Plus Nucleic Acid Extractor and 101 cartridge, DNA concentration was measured using NanoDrop™ 2000 (Thermo Scientific™) and Qubit (Thermo Scientific™).

Exome sequencing

Strictly following manufacturer's instructions, library preparation was performed using the Twist Library Preparation EF Kit and the Twist Universal Adapter System - TruSeq Compatible, 96 Samples Plate A-D. Target enrichment was achieved using the Twist Core Exome and the Twist Exome 2.0 from Twist Bioscience. Paired-end next generation sequencing was performed using the NovaSeq S4 Reagent Kit v1.5 on an Illumina NovaSeq 6000 platform. Coverage of more than 20x has been achieved in 99.7% of target sequences. Analysis of the raw data was performed using the software Varfeed (Limbus, Rostock), the variants (SNVs and CNVs) were annotated using the software Varvis (Limbus, Rostock) including MT-DNA analysis which however was not available for all exomes at the time of initial data analysis.

Table S1: Symptoms on request of genetic diagnostics, suspected diagnosis: HSP: hereditary spastic paraplegia, SCA: spinocerebellar ataxia, year of first whole exome sequencing (WES), targeted testing of repeat expansions in genes prior to this study, identified diagnosis in this study, identified genetic variant/repeat expansion, inheritance of identified disease; AD: autosomal-dominant, AR: autosomal-recessive

**Patient**	**1**	**2**	**3**	**4**	**5**	**6**	**7**	**8**
**Symptoms on diagnostic request**	cerebellar ataxia, spasticity, periphery neuropathy	cerebellar ataxia, sensory neuropathy	ataxia, tremor	ataxia, cognitive impairment	cerebellar ataxia	cerebellar ataxia, vertigo	spastic-atactic gait, vertigo	spastic-atactic gait, vertigo, tremor
**Suspected diagnosis**	HSP, Friedreich ataxia	SCA	SCA	SCA	SCA	SCA	Hereditary ataxia	Hereditary ataxia, HSP
**Year of first WES analysis**	2019	2021	2022	2017	2019	2020	2023	2022
**Previously targeted testing (genes)**	*FXN*	*ATXN1, ATXN2, ATXN3, CACN1A, ATXN7, ATXN8OS, ATXN10, PPP2R2B, TBP, FMR1*	*ATXN1, ATXN2, ATXN3, CACN1A, ATXN7, ATXN8OS, ATXN10, PPP2R2B, TBP, FGF14, BEAN1, NOP56, DAB1, RFC1, ATN1, FXN, FMR1*	*NOTCH3, ATXN1, ATXN2, ATXN3, CACN1A, ATXN7, ATXN8OS, ATXN10, PPP2R2B, TBP, DRPLA*	*ATXN1, ATXN2, ATXN3, CACN1A, ATXN7, ATXN8OS, ATXN10, PPP2R2B, TBP, FMR1*	*ATXN1, ATXN2, ATXN3, CACN1A, ATXN7, ATXN8OS, ATXN10, PPP2R2B, TBP, FMR1*	None	None
**Identified diagnosis**	MT-*ATP6*-associated mitochondriopathy	Spinocerebellar ataxia type 1 (SCA1)	Huntington's Disease (HD)	Myotonic dystrophy type 2 (DM2)	Spinocerebellar ataxia type 37 (SCA37)	Cerebellar ataxia, neuropathy, and vestibular areflexia syndrome (CANVAS)	Very-late-onset Friedreich ataxia (VLOFA)	None
**Disease-causing variant/ repeat expansion**	m.9185T>C in *MT-ATP6*	CAG[44]in *ATXN1*, heterozygous	CAG[47-52] in *HTT*, heterozygous	CCTG[100-2840] in *CNBP*, heterozygous	ATTTT[141]ATTTC[97] ATTTT[10] in *DAB1*, heterozygous	AAGGG[862-891]/ AAGGG[1010-1159] in *RFC1*	GAA[97-122]/ GAA[773-1088] in *FXN*	None
**Inheritance**	AD	AD	AD	AD	AD	AR	AR	Unknown

Short-read genome sequencing

For short-read genome library preparation was done using the Twist Library Preparation EF Kit1, 2.0 and Twist Universal Adapter System - TruSeq Compatible, 96 Samples Plate A-D strictly following manufacturer's instructions and SOPs. Paired-end next generation sequencing (2x 150 bp) was then performed on a NovaSeq 6000 Instrument using an S4 Reagent Kit (Illumina, Inc., San Diego, CA, USA). Raw data analysis was performed using Dragen4.0 implemented in the Emedgene software and variants were scored and prioritized using the Emedgene software (Illumina, Inc., San Diego, CA, USA). We performed a genome wide detection of CNVs, SVs and repeat expansion with DragenCNV4.0, Dragen-Manta4.0 and ExpansionHunter implemented in Emedgene.

ExpansionHunter

In addition to the implemented version in Emedgene, ExpansionHunter version v4.0.2 was run separately according to the developer's instructions using the variant catalogs from Broad Institute [2] (Online Resource 2 and 3). Repeat expansions were considered to be pathogenic above the pathogenic threshold according GeneReviews [3] or Stripy STRs database [4] (Online Resource 4).

Long-read genome sequencing

DNA integrity was measured using a 4150 TapeStation System (Agilent) with the Genomic DNA ScreenTape assay and the TapeStation analysis software [5]. DNA (3 µg) was prepared using the Ligation Sequencing Kit (SQK-LSK114, Oxford Nanopore Technologies, ONT) according to the manufacturer's instructions with the following adjustments: Incubation time of the end-prep reaction was increased to 15 min at 20 °C and 15 min at 65 °C. Incubation time of adapter ligation reaction was adjusted to 30 min at room temperature. Each library (300 ng) was loaded onto a PromethION flow cell type R10.4.1 and sequenced on a P2Solo device (ONT). The flow cell was washed once or twice using Wash Kit EXP-WSH004 (ONT) according to the manufacturer's instructions. After each washing step, another 300 ng of DNA library was loaded onto the flow cell. Raw sequencing data (fast5/pod5 files) was basecalled and mapped against the reference human genome hg38 using dorado 0.3.4, 0.5.0 or 0.5.3 (ONT) using a methylation-aware super accuracy basecalling model with enabled read splitting and adapter trimming. STR genotyping (straglr) was performed using the human variation workflow from epi2me-labs (ONT) and relevant STR expansions were inspected manually using IGV (see Online Resource 5).

Extended discussion on repeat expansion detection in short-read sequencing data

Mitochondriopathy and repeat expansions in coding regions, as *ATXN1* and *HTT*, could be identified readily in short-read exome data using bioinformatic tools, which was already shown by van der Sanden et al. [6]. However, exome sequencing does not enable the detection of variants and repeat expansions in non-coding regions, such as CANVAS and SCA37. Rudaks et al. [7] suggested to perform long-read or short-read WGS with bioinformatic tools for repeat expansion detection as first genetic test in ataxia patients without a suspected specific disease or without a known genetic disorder in the family. In this study, we found indications of at least hints of the four repeat expansions in non-coding regions in short-read WGS. To this end, different challenges were identified.

First, the large repeat expansion in the gene *CNBP* was only estimated to be pathogenic in the separate ExpansionHunter calculation, but not in the evaluation software with integrated ExpansionHunter. Estimation of very large expansions required inclusion of the off-target function (see Dolzhenko et al. [8]). As the evaluation software is not open source, we do not know, why the large repeat expansion was not detected by ExpansionHunter implemented in the evaluation software.

Second, the pathogenic motifs in the genes *DAB1* and *RFC1* were not distinguished from physiological motifs using ExpansionHunter and might only be identified by visual IGV inspection.

Third, in case of the biallelic repeat expansion in *FXN*, only a monoallelic repeat expansion was detected, consistent with Ibanez et al. in 2022 [9]. These findings highlight the limitations of ExpansionHunter in detecting pathogenic motifs and biallelic expansions.

Overall, the feasibility of repeat expansion detection in short-read WGS using ExpansionHunter was verified only for a few loci in large cohorts [9]. In a study 32 pathogenic repeat expansions in eight genes were identified in short-read WGS data in 380 patients, but the common SCA27B could not be detected [10]. Another study demonstrated the feasibility of detecting potential pathogenic repeat expansions in *FGF14* and *RFC1* in short-read WGS by lowering the threshold value for detecting potential repeat expansions, but repeat expansions in genes like *DAB1* were not detected [11]. The authors also observed an improvement of the diagnostic outcomes through visual IGV inspection.

In contrast to short-read sequencing, precise genotyping of repeat expansions is possible with long-read sequencing, which was also shown in this study. Long-read sequencing can detect repeat expansions with a high sensitivity and has the ability to determine even large repeat expansions including complex motifs and interruptions, and the identification of non-repeat associated variants on the second allele. These are all relevant for prediction of pathogenicity, age of onset and severity of the disease [12, 13]. Nonetheless, somatic variability regarding repeat size and repeat motif is still a challenge for exact genotyping by long-read sequencing [14]. Long-read WGS is yet expensive, but targeted long-read sequencing, as the nanopore Cas9-targeted sequencing panel developed by Erdman et al. [15] can be a more cost-effective approach, albeit limited to the included genes and not enabling re-evaluations.

References

[1] Robinson JT, Thorvaldsdóttir H, Wenger AM, Zehir A and Mesirov JP (2017) Variant review with the integrative genomics viewer. Cancer Res 77:e31–e34. https://doi.org/10.1158/0008-5472.CAN-17-0337

[2] Broad Institute, 'https://github.com/broadinstitute/str-analysis/tree/main/str_analysis/variant_catalogs'. Accessed on 2023-06.

[3] Adam MP, Bick S, Mirzaa GM, et al., editors., GeneReviews (®), 1993-2025.

[4] Halman A, Dolzhenko E and Oshlack A (2022) STRipy: A graphical application for enhanced genotyping of pathogenic short tandem repeats in sequencing data. Hum Mut 43:859–868. https://doi.org/10.1002/humu.24382

[5] Faust H, Duffek P, Hentschel J and Popp D (2024) Evaluation of automated magnetic bead-based DNA extraction for detection of short tandem repeat expansions with nanopore sequencing. J Clin Lab Anal 38:e25029. https://doi.org/10.1002/jcla.25029

[6] Van der Sanden BPGH, Corominas J, de Groot M et al (2021) Systematic analysis of short tandem repeats in 38,095 exomes provides an additional diagnostic yield. Genet Med 23:1569–1573. https://doi.org/10.1038/s41436-021-01174-1

[7] Rudaks LI, Yeow D, Ng K, Deveson IW, Kennerson M and Kumar KR. (2024) An update on the adult-onset hereditary cerebellar ataxias: Novel genetic causes and new diagnostic approaches. Cerebellum 23:2152–2168. https://doi.org/10.1007/s12311-024-01703-z

[8] Dolzhenko E, van Vugt JJFA, Shaw RJ et al (2017) Detection of long repeat expansions from PCR-free whole-genome sequence data. Genome Res 27:1895–1903. https://doi.org/10.1101/gr.225672.117

[9] Ibañez K, Polke J, Hagelstrom RT et al (2022) Whole genome sequencing for the diagnosis of neurological repeat expansion disorders in the UK: A retrospective diagnostic accuracy and prospective clinical validation study. Lancet Neurol 21:234–245. https://doi.org/10.1016/S1474-4422(21)00462-2

[10] Yau WY, Sullivan R, O'Connor E et al (2025) Diagnostic yield and limitations of whole-genome sequencing for hereditary cerebellar ataxia. Brain Commun 7:fcaf188. https://doi.org/10.1093/braincomms/fcaf188

[11] Rafehi H, Fearnley LG, Read J et al (2025) A prospective trial comparing programmable targeted long-read sequencing and short-read genome sequencing for genetic diagnosis of cerebellar ataxia. Genome Res 35:769–785. https://doi.org/10.1101/gr.279634.124

[12] Stevanovski I, Chintalaphani SR, Gamaarachchi H et al (2022) Comprehensive genetic diagnosis of tandem repeat expansion disorders with programmable targeted nanopore sequencing. Sci Adv 8:eabm5386. https://doi.org/10.1126/sciadv.abm5386

[13] Rajan-Babu IS, Dolzhenko E, Eberle MA and Friedman JM. (2024) Sequence composition changes in short tandem repeats: Heterogeneity, detection, mechanisms and clinical implications. Nat Rev Genet 25:476–499. https://doi.org/10.1038/s41576-024-00696-z

[14] Zou J, Wang F, Gong Z et al (2023) A Chinese SCA36 pedigree analysis of *NOP56* expansion region based on long-read sequencing. Front in Genet 14:1110307. https://doi.org/10.3389/fgene.2023.1110307

[15] Erdmann H, Schöberl F, Giurgiu M et al (2023) Parallel in-depth analysis of repeat expansions in ataxia patients by long-read sequencing. Brain 146:1831–1843. https://doi.org/10.1093/brain/awac377
