# Supplementary figures and images for: Clinical and genetic diagnostic challenges in presumed hereditary ataxia
Source: J Neurol. 2026 Mar 23;273(4):225. doi: 10.1007/s00415-026-13756-7 (PMC13009057; doi:10.1007/s00415-026-13756-7)

## Patient 2 - *ATXN1*

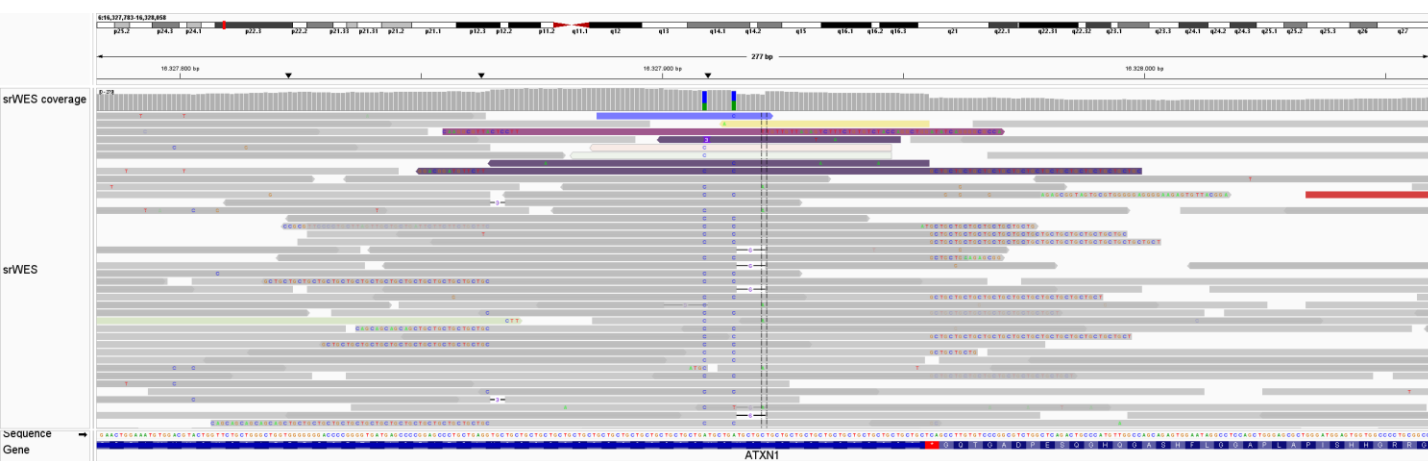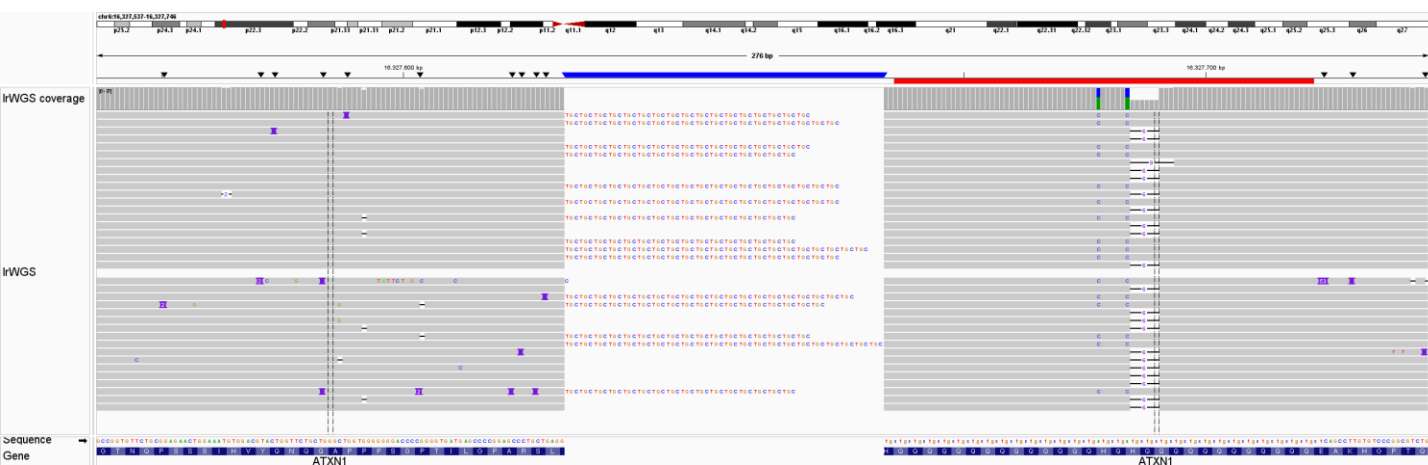

Patient 3 - *HTT*

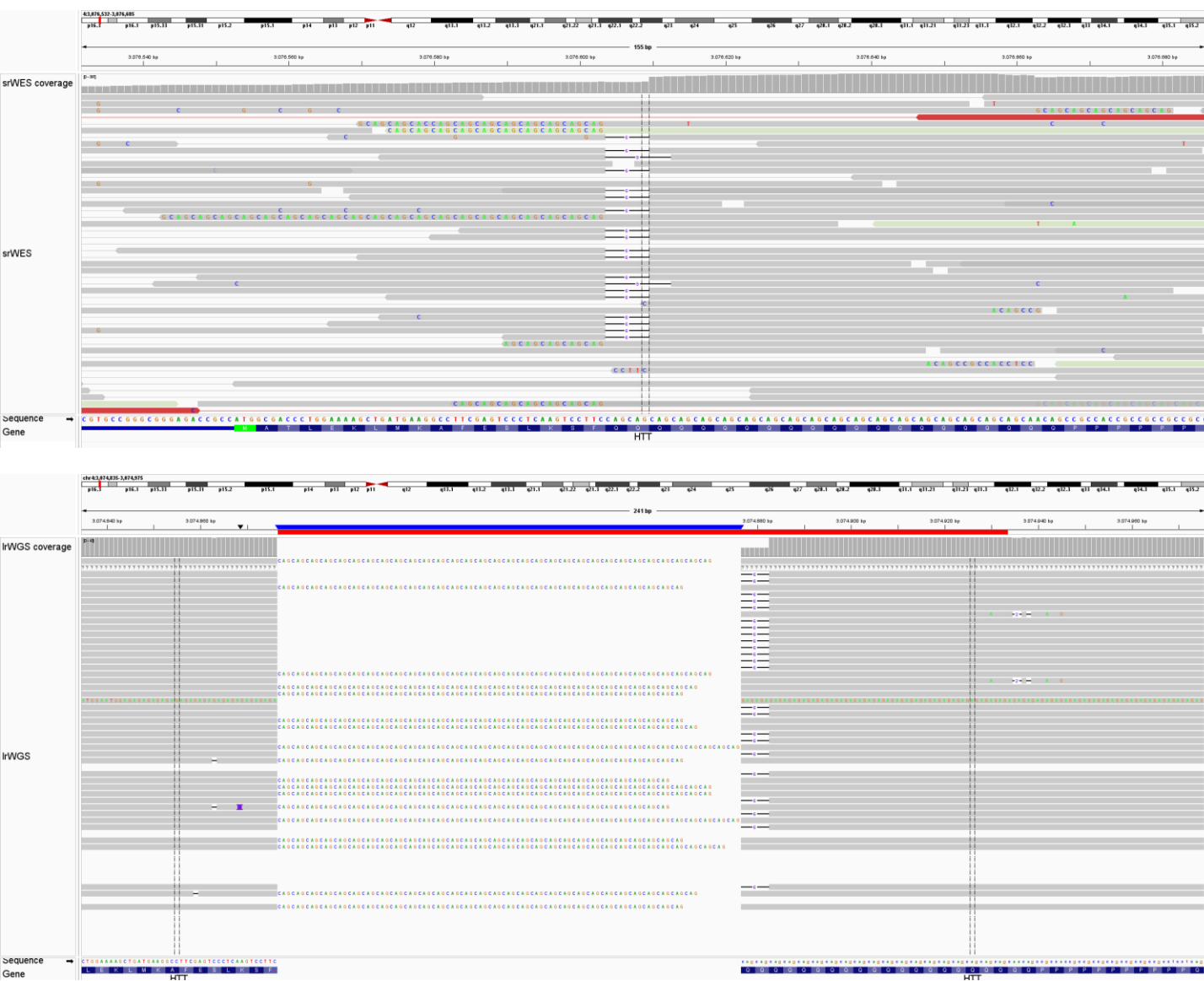

# Patient 4 - *CNBP*

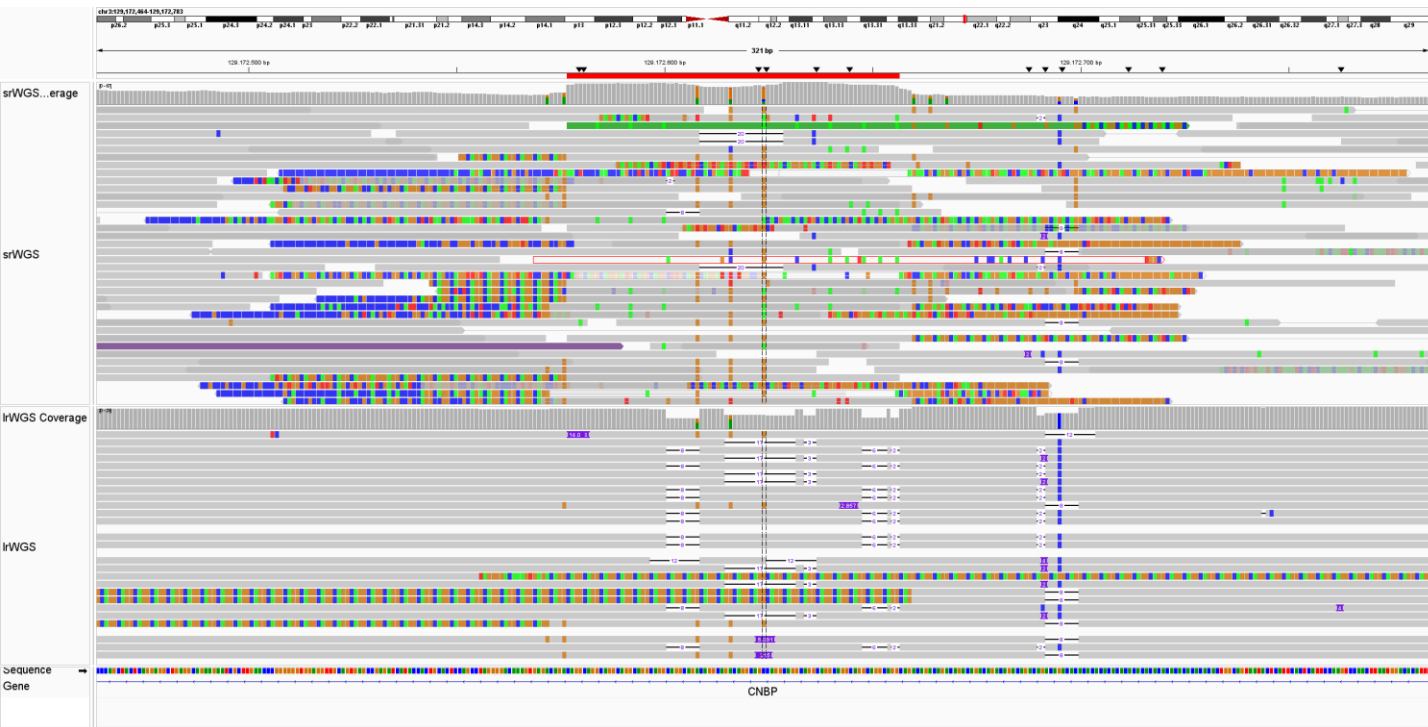

# Patient 5 – *DAB1*

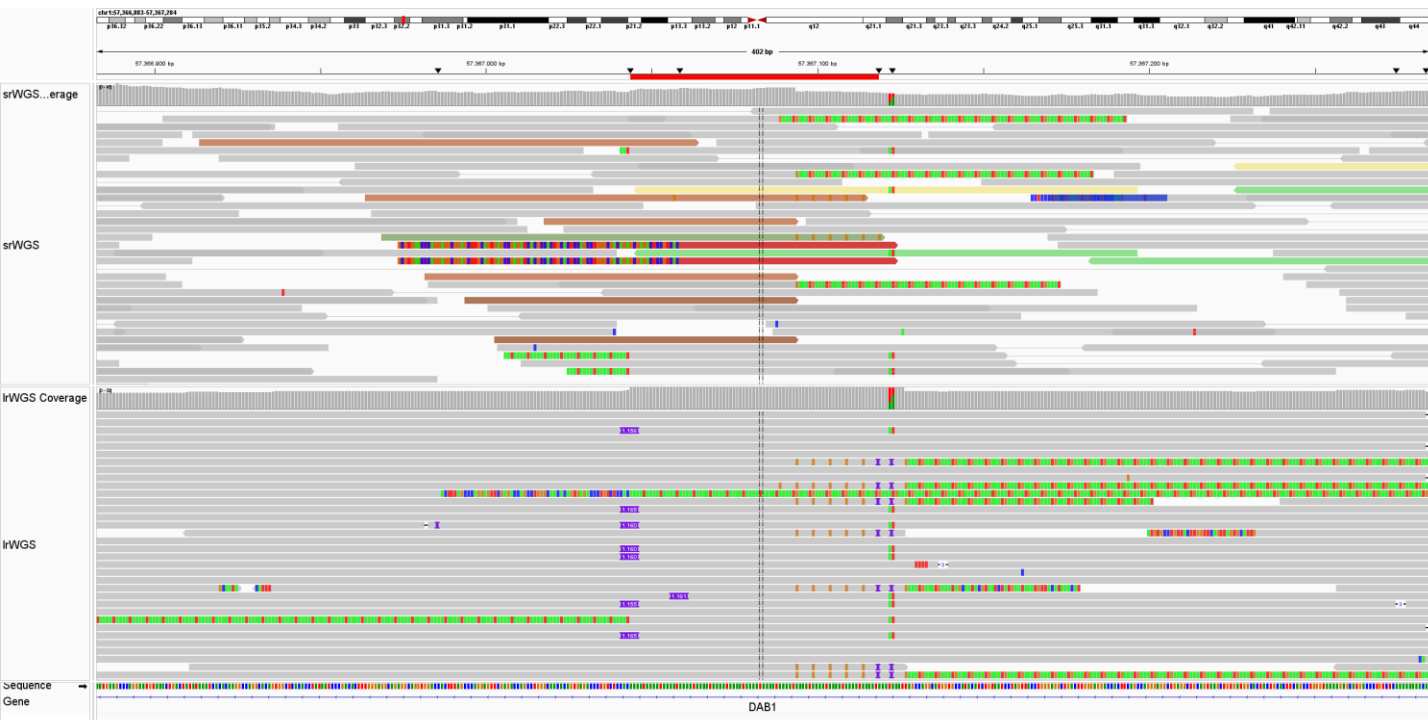

# Patient 6 – RFC1

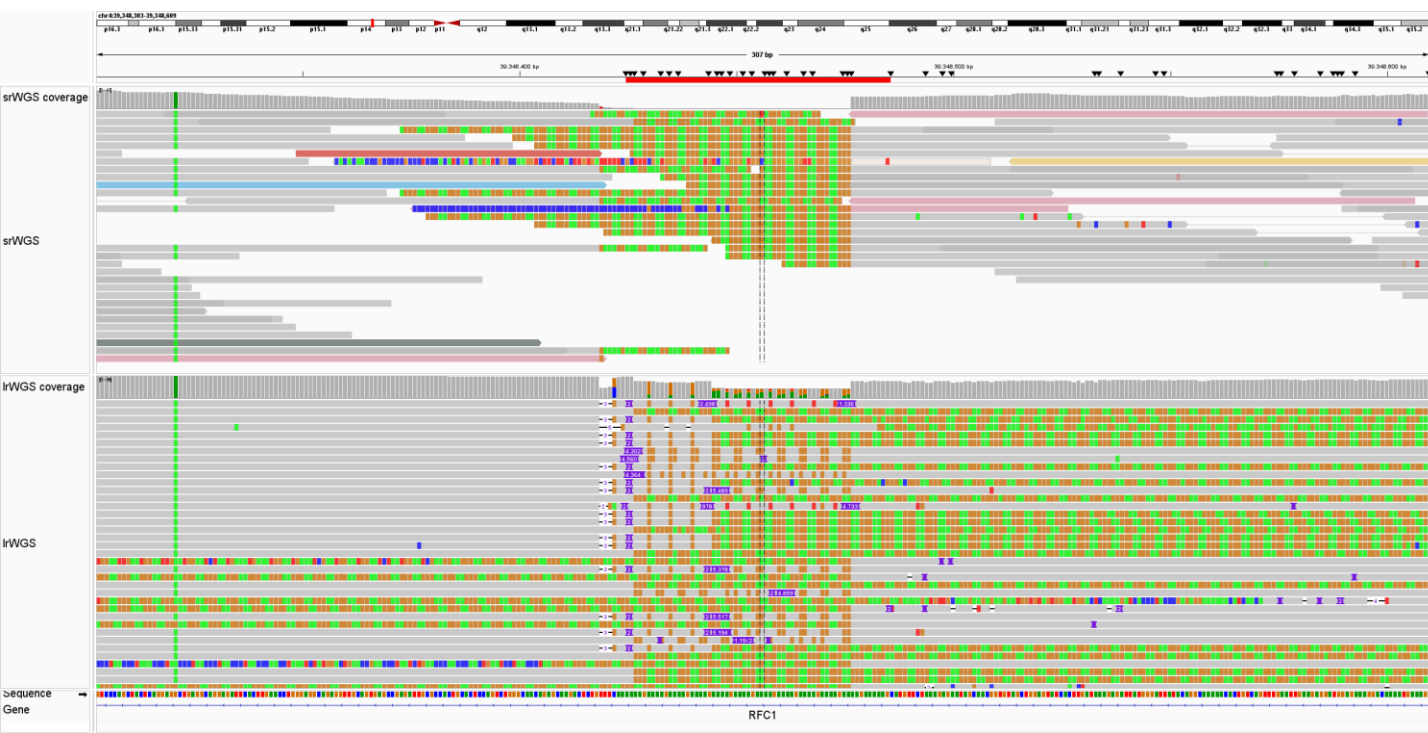

# Patient 7 – FXN

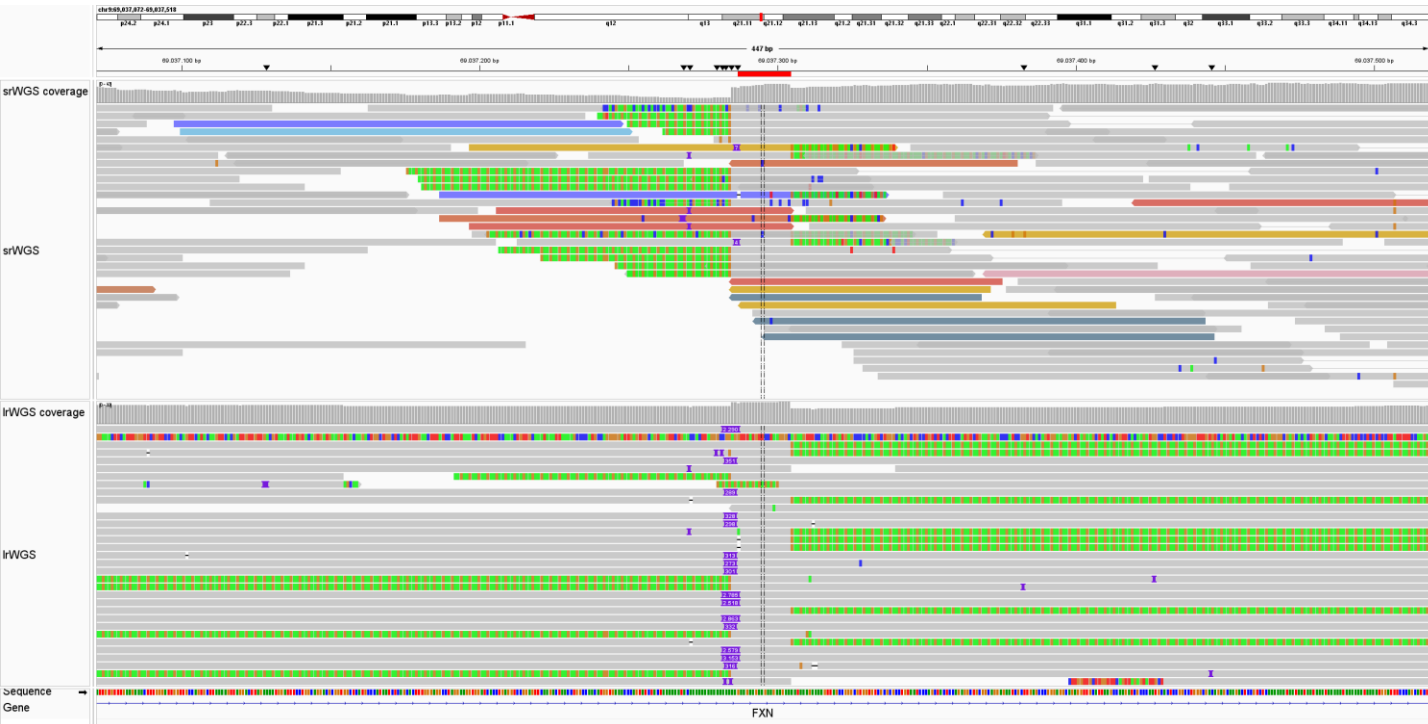

Supplement: Supplementary file 5 — Online Resource 5: IGV screenshots of relevant STR expansions. Supplementary file5 (PDF 900 KB) [file 415_2026_13756_MOESM5_ESM.pdf]
